# MetaScreener: a robust dual-mode framework for directional prioritization of actionable signatures through multi-dataset and multi-approach integration

**DOI:** 10.1186/s12967-026-08019-y

**Published:** 2026-03-17

**Authors:** Dingkang Zhao, Gaoxiang Zhao, Minghui Yao, Jianxiong Wu, Zhaoyuan Fang

**Affiliations:** 1 Department of Colorectal Surgery and Oncology, the Second Affiliated Hospital, and Center for Biomedical Systems and Informatics, Zhejiang University-University of Edinburgh Institute (ZJU-UoE Institute), Zhejiang University School of Medicine, Zhejiang University, Hangzhou, Zhejiang 310000 China; 2 Edinburgh Medical School, Biomedical Sciences, College of Medicine and Veterinary Medicine, University of Edinburgh, Edinburgh, Scotland EH8 9YL UK; 3 Key Laboratory of Tropical Translational Medicine of Ministry of Education, School of Basic Medicine and Life Sciences, Hainan Medical University, Haikou, Hainan 571199 China

**Keywords:** Signature directionality, Multi-dataset integration, Multi-approach integration, Directional prioritization, Drug discovery

## Abstract

Reliably quantifying the directionality of signatures from noisy omics data remains challenging, hindering their widespread applications in biomedical research and drug development. To address this, we developed MetaScreener, a dual-mode framework (DiffMetaScreener and CorMetaScreener) that integrated multiple datasets and a large number of analysis pipelines (>4, 000) for robust quantification of activating and inhibiting signals in gene signatures. Supervised application of MetaScreener on 9 colorectal cancer (CRC) training datasets prioritized positive and negative Wnt signaling signatures, respectively. Robustness benchmarking against random perturbations, including extreme noises, revealed that MetaScreener outperformed conventional approaches. Independent validation on 22 CRC datasets and a massive-scale single-cell dataset further confirmed the accuracy of the prioritized signatures. In the case study, parthenolide was discovered and experimentally validated as a dual inhibitor targeting both Wnt signaling and CRC metastasis. Collectively, MetaScreener demonstrates as a reliable and powerful tool for directional prioritization of both functional and actionable gene signatures.

**Supplementary Information:**

The online version contains supplementary material available at 10.1186/s12967-026-08019-y.

## Introduction

A big and fundamental question of biology remains hunting for the functional determinants of phenotypes [1–6], which act inherently in a directional and causal manner [7–9]. Hence, directionality naturally underlies the widely employed perturbation paradigms, ranging from CRISPR-guided gene knock-in/out [10, 11] to small ligand-mediated target activation/inhibition [12, 13]. For decades, however, sharp discrepancies persist between the actionable and directed ‘wet’ side and the computational and associative ‘dry’ side, despite the many data resources established and algorithms developed. Most of the existing functional gene set databases, such as Gene Ontology (GO) [14], and MSigDB [15, 16], serve as collections of functional terms and gene signatures without rigorously verified directions. Hallmark [17], a widely used non-redundant subset of MSigDB, remains partially non-directional. KEGG [18] and Cell Collective [19] include directed edges within their pathway models, which as a whole are not directed for pathway-level activity evaluation. Quantitative methods, such as GSVA [20], ssGSEA [21], Z-score [22], PLAGE [23], AUCell [24], UCell [25], Singscore [26], and decoupleR [27], are either direction-unaware or simply assuming a positive direction of the functional terms analyzed. These lacking-of-direction issues significantly restrict the explainability and translatable application of functional gene signatures.

Furthermore, additional challenges originate from the prominent dispersion of high-throughput genomics data. On the one hand, complex biological samples embrace both intrinsic and extrinsic stochasticity [28–30]. Intrinsic sources of variation typically include genetic alterations [31], epigenetic reprogramming [32], transcriptional and translational noise [33], and biochemical oscillations [34, 35]. Extrinsic sources of variation can come from microenvironmental factors attributable to ligands, extracellular matrix compositions, neighboring cell types, microorganisms, as well as other spatial or non-spatial effects in tissues [36–38]. On the other hand, technical variability and noises are substantial in recent transcriptomics technologies such as single-cell RNA sequencing (scRNA-seq) and spatial transcriptomics [39, 40]. To complicate matters further, these biological and technical variations frequently intertwine, rendering the quest for optimal models or solutions, a central challenge in applying machine learning to modern oncology and drug discovery, largely controversial or even unattainable [41–43].

To address these challenges, we develop MetaScreener, a comprehensive integration framework for directional prioritization of functional gene sets and signatures. For directionality enforcement, it accepts training datasets with either discrete labels or continuous states, and extracts directionality measures through differential (DiffMetaScreener) and correlative (CorMetaScreener) modes, respectively. To achieve robustness against the excessive noises in omics data, MetaScreener integrates a large number of pipelines or algorithms and operates in a multi-dataset integration manner. Moreover, it also supports both supervised and unsupervised analyses. We rigorously validate its statistical power for weak signals in noisy and incomplete data, and systematically evaluate its accuracy and reliability in 31 colorectal cancer (CRC) datasets, focusing on the heterogeneity of Wnt/β-catenin signaling in CRC. Finally, we apply MetaScreener to prioritize a candidate drug that suppresses Wnt activity and inhibits CRC metastasis, followed by experimental validation.

## Results

### A dual-mode framework for signature screening through directionality quantification

To reliably quantify the bidirectional activation and inhibition properties of signatures in specific biological contexts, we need to develop a systematic framework to satisfy the compilation, quality control, directional scoring, and context-specific evaluation of candidate signatures.

**Fig. 1:**
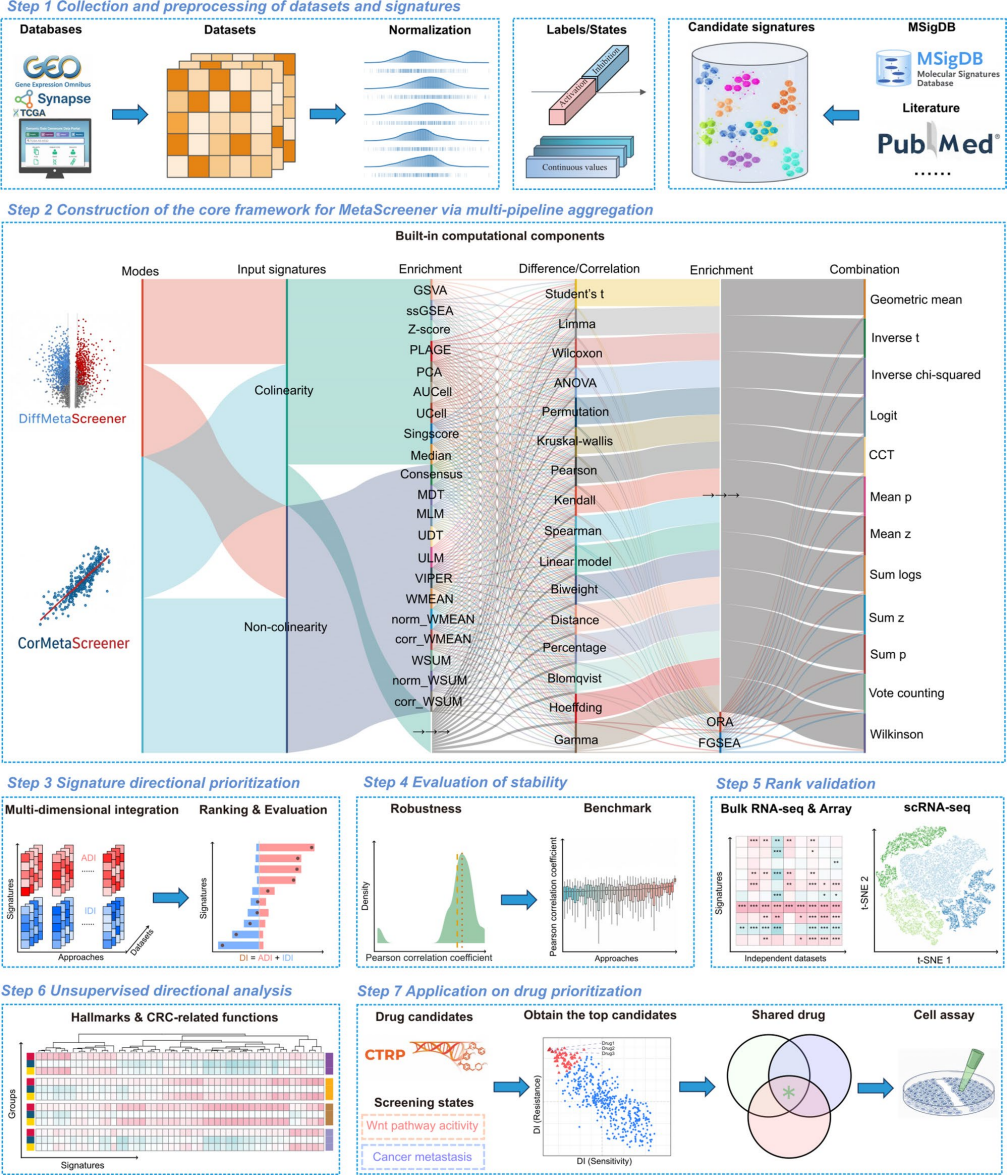
Analysis workflow with MetaScreener. Step 1. Collection and preprocessing of datasets and signatures. Omics datasets such as transcriptomics, epigneomics, and proteomics are all applicable, given that the signatures and the datasets are matching and that all datasets are appropriately calibrated. Sample information can be either discrete labels or continuous states such as signature scores. Candidate signatures can be compiled from public databases or literature. Step 2. Construction of the core framework for MetaScreener via multi-pipeline aggregation. Two distinct modes (DiffMetascreener and CorMetaScreener) are available. Each mode has an option for signature collinearity assessment, and together integrates 21 unsupervised and 2 supervised enrichment analysis methods, 6 differential analysis and 10 correlative analysis approaches, and 12 meta-analysis algorithms for *p*-value combination. Step 3. Signature directional prioritization. This step involves a multidimensional integration across approaches and datasets, and in both directions of signatures (ADI and IDI). Ranking and evaluation are conducted based on the final scores (DI). ADI: activation directionality indices; IDI: inhibition directionality indices; DI: directionality indices. Step 4. evaluation of framework stability. This step primarily involves a systematic evaluation of robustness and validity. Step 5. after obtaining the directional ranking of the signatures, validate the ranking in independent RNA-seq, microarrays, and single-cell RNA-seq datasets. Step 6. exploring the empirical consistency of hallmarks and CRC-related functions under conditions where the directionality of signatures is unknown. Step 7. this framework can be applied in various scenarios including drug discovery. After defining the multiple screening states and candidate drugs, the framework enables combinatory and directional ranking of compound candidates, which could be experimentally validated through cell-based assays

In this framework, the initial step involved collection of multiple datasets and candidate signatures (Step 1 in Fig. 1). These datasets were formatted as numerical matrices, which could encompass various biological omics data such as transcriptomics, epigenomics, proteomics, and metabolomics. Following recalibration, each sample within these datasets was annotated with either binary ‘activation/inhibition’ labels or a set of continuous quantities such as state variables. These continuous variables could represent the expression level of a specific gene, the activity level of a particular pathway, observed clinical features associated with patients, or even user-defined numerical values corresponding to the samples. Simultaneously, candidate signatures were gathered, sourced from databases (MSigDB, Enrichr, HMDB, or PathCards), literature compilations, custom definitions, or *de novo* gene sets derived from computational methods. Overall, this framework was designed for high flexibility and to address diverse research needs.

The subsequent step established the core computational framework (Step 2 in Fig. 1), which could operate in two distinct modes: DiffMetaScreener and CorMetaScreener. The former required binary ‘activation/inhibition’ labels, while the latter utilized continuous variables. Following the mode selection, potential collinearity was assessed among candidate signatures to guide appropriate enrichment method choices. The framework integrated comprehensive analytical pipelines, with DiffMetaScreener combining the 23 enrichment methods with 6 differential analyses (yielding 138 differential enrichment pipelines) and CorMetaScreener combining the same 23 enrichment methods with 10 correlative analyses (yielding 230 correlative enrichment pipelines), collectively offering 368 distinct functional profiling strategies for comprehensive biological interpretation. Finally, 12 meta-analysis algorithms were employed to integrate those statistical outputs from above computations, thereby rigorously ensuring framework stability.

The third step of the framework involved mathematically processing the raw integrated results to derive directionality indices (DI) for screening target signatures (Step 3 in Fig. 1). The one-tailed statistical outputs produced consistently positive activation directionality indices (ADI) and negative inhibition directionality indices (IDI) through -log_10_-transformation and log_10_-transformation, respectively. These values were then aggregated to obtain the final ADI and IDI, the summation of which was DI.

Subsequently, the robustness and validity of the framework was assessed (Step 4 in Fig. 1), with additional validation of the directionality was performed using independent datasets (Step 5 in Fig. 1) and in connection with established biological signatures (Step 6 in Fig. 1). Finally, the framework also proved effective in the application of combinatory and actionable drug screening (Step 7 in Fig. 1). Overall, the ensemble-based computational framework enhances the reliability and generalization capability of signature evaluation by integrating outputs from multiple datasets and algorithms [44].

### Robust identification of directional Wnt pathway signatures in colorectal cancer

**Fig. 2:**
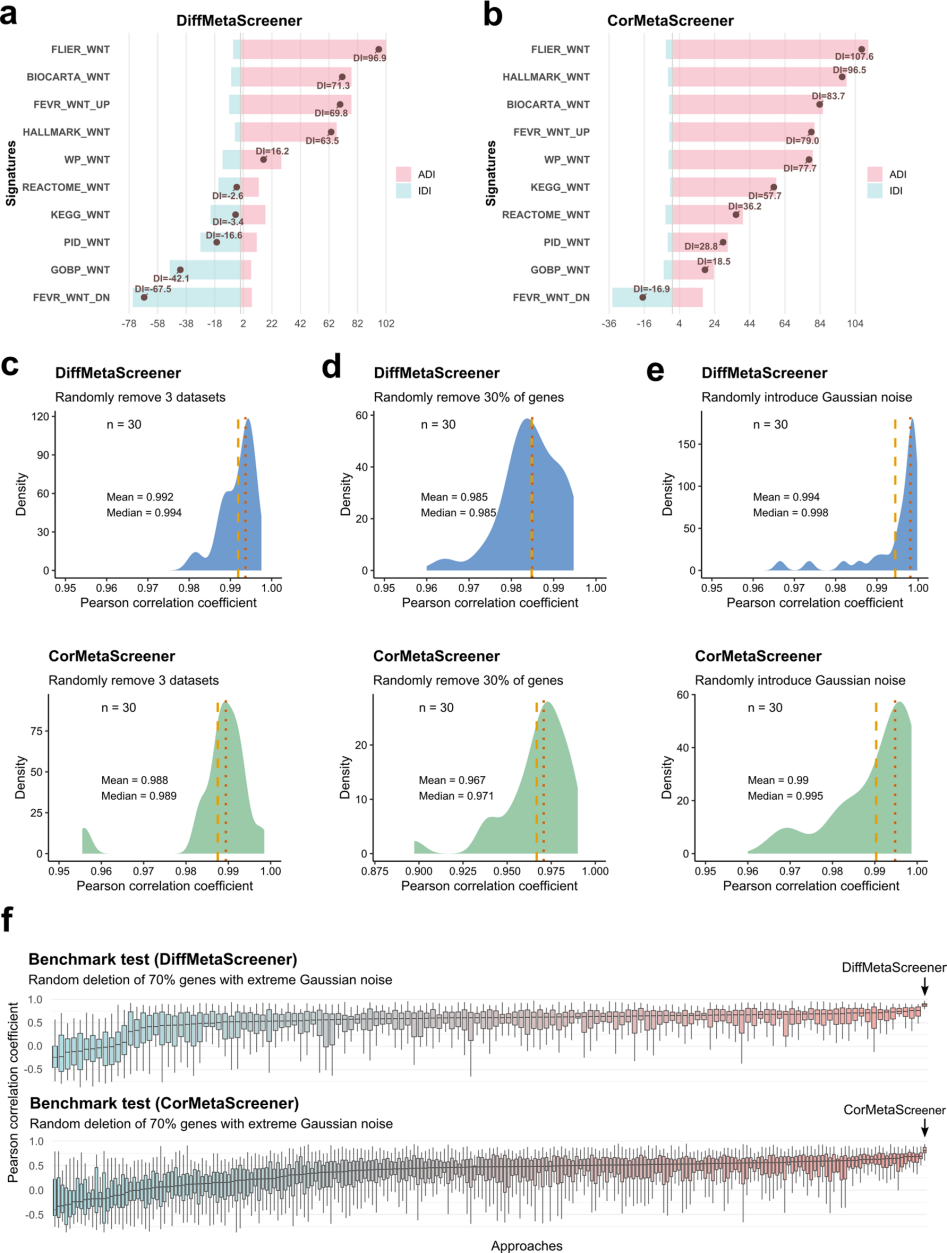
Validity, robustness, and benchmarking of MetaScreener in CRC. (**a**) Directional bar graph of 10 Wnt pathway candidate signatures with DiffMetaScreener. (**b**) Directional bar graph of 10 Wnt pathway candidate signatures with CorMetaScreener. (**c**) Robustness test by randomly removing 3 training datasets. Repeat 30 times. (**d**) Robustness test with 30% of genes randomly removed from each training dataset. Repeat 30 times. (**e**) Robustness test by randomly introducing Gaussian noise (mean = 0, standard deviations ranging from 0.05 - 0.2) for each training dataset. The proportion of noise ranged from 0% to 100%. Repeat 30 times (15 times each for additive and multiplicative noise). (**f**) Benchmarking of MetaScreener with all individual algorithms. Random deletion of 70% of the genes was accompanied by the introduction of strong noise (mean = 1, standard deviations ranging from 1 - 2), and the proportion of noise was 50% − 100%. Repeat 30 times (15 times each for additive and multiplicative noise)

To evaluate the validity of MetaScreener, we took advantage of the Wnt/β-catenin pathway, which serves as a well-known driver in a significant fraction of CRC. In the CMS subtyping system, it is the CMS2 subtype that exhibited hyperactivation of the Wnt pathway compared to other subtypes [45]. Similarly, in the iCMS subtyping system, the iCMS2 subtype demonstrated significantly higher Wnt activity than iCMS3 [46]. In our research, we curated 9 high-quality CRC datasets (Table S1-2) and employed MetaScreener to quantify the directionality of 10 candidate Wnt pathway signatures (Table S3). As expected, both DiffMetaScreener and CorMetaScreener modes consistently captured directional biological signals, with ‘FLIER_WNT’ best signature of Wnt pathway activation and ‘FEVR_WNT_DN’ for inhibition (Figs. 2a and 2b). Additionally, there are interesting differences between the two modes. DiffMetaScreener was more adept at detecting balanced signals, with half of the 10 candidate signatures showing positive DI and the other half negative. On the other hand, CorMetaScreener appeared to identifying Wnt pathway activation signals, with 9 signatures exhibiting positive directionality. Such differences could be attributed to the underlying algorithmic structures and the nature of input labels (discrete ‘activation/inhibition’ labels or continuous variables). Consistent results were also observed in a more qualitative assessment of directionality, further corroborating the above findings from our framework (Fig. S1a-b).

Through multi-dataset and multi-approach integration, MetaScreener was expected to be highly robust. To verify this, we sought to determine the influence of data and/or method perturbation. On the CRC datasets, we introduced various types of perturbation, including random removal of datasets or genes, Gaussian noises, and parameter dropouts. MetaScreener demonstrated consistent performance and exhibited strong resilience to both data loss and noise incorporation (Fig. 2c–e). It also revealed minimal dependence on specific pipeline parameters (Fig. S1c-f). Furthermore, under extreme data loss and strong noise, MetaScreener significantly outperformed all conventional single-dataset and single-enrichment approaches (Fig. 2f–g and Fig. S2).

### Large-scale independent validation of directional Wnt pathway signatures

**Fig. 3:**
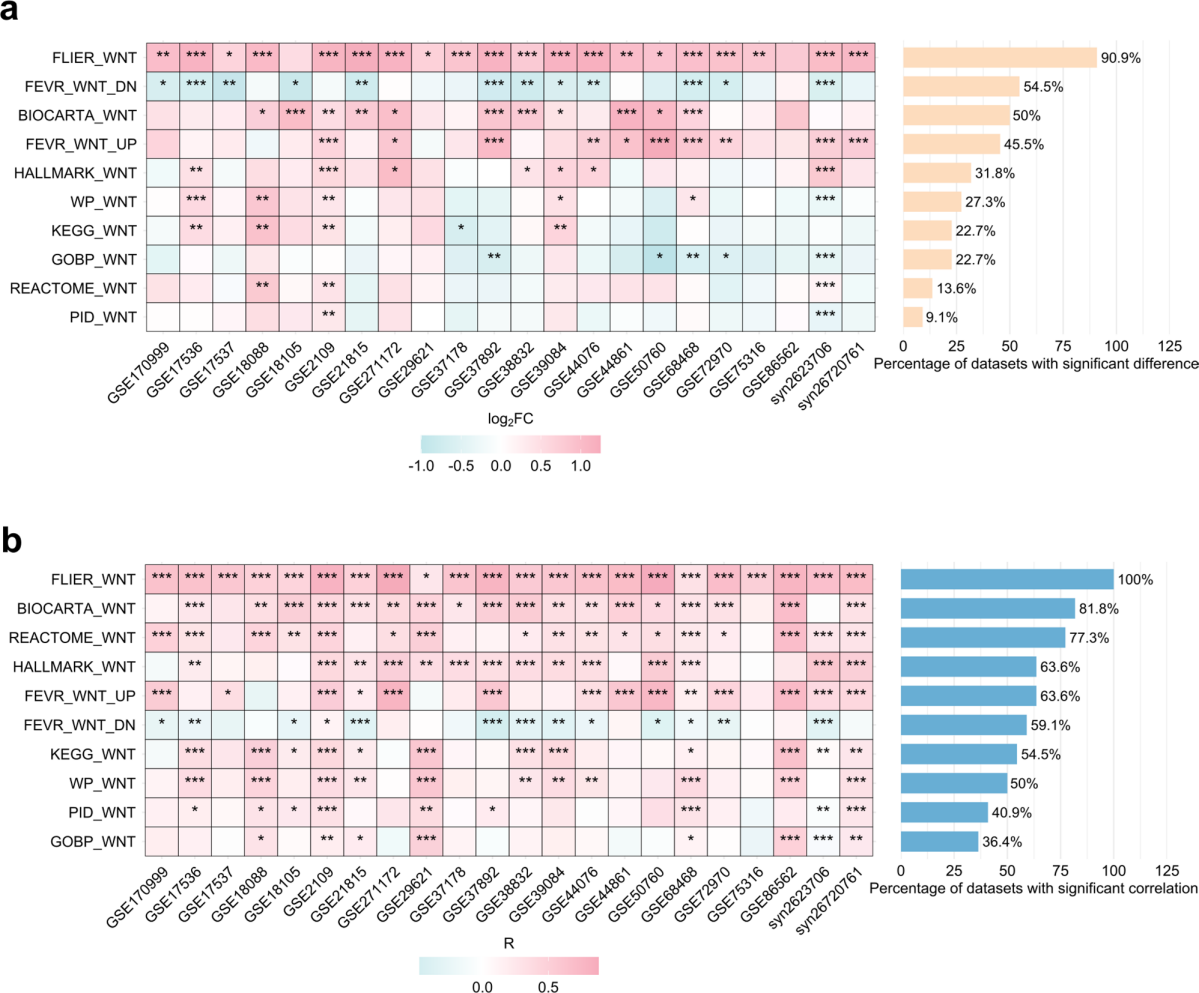
Analyses of 22 CRC independent validation datasets. (**a**) Evaluation of signature directionality based on differential enrichment analysis. * *p*-value < 0.05, ** *p*-value < 0.01, *** *p*-value < 0.001. (**b**) Evaluation of signature directionality based on correlative enrichment analysis. * *p*-value < 0.05, ** *p*-value < 0.01, *** *p*-value < 0.001

To validate the accuracy of MetaScreener, we compiled 22 independent CRC datasets (Table S1-2) with CMS classification and iCMS signature scores, and tested the performance of the 10 candidate Wnt pathway signatures (Table S3). Under the discrete CMS classification and the DiffMetaScreener mode, the ‘FLIER_WNT’ signature demonstrated superior performance, showing significantly higher Wnt activity in CMS2 subtypes compared to other subtypes across 20/22 datasets (Fig. 3a). The ‘FEVR_WNT_UP’, ‘BIOCARTA_WNT’, and ‘FEVR_WNT_DN’ signatures also exhibited consistently good performance, achieving statistical significance in 10/22, 11/22, and 12/22 datasets respectively (Fig. 3a), matching the previous training data well (Fig. 2a). These findings were further in line with the quantitative screening results from the CorMetaScreener mode (Figs. 3b and 2b). Collectively, these validation results confirmed the accuracy and reliability of MetaScreener in identifying signatures and quantifying directionality.

### Evaluation of directional Wnt pathway signatures at a single-cell resolution

**Fig. 4:**
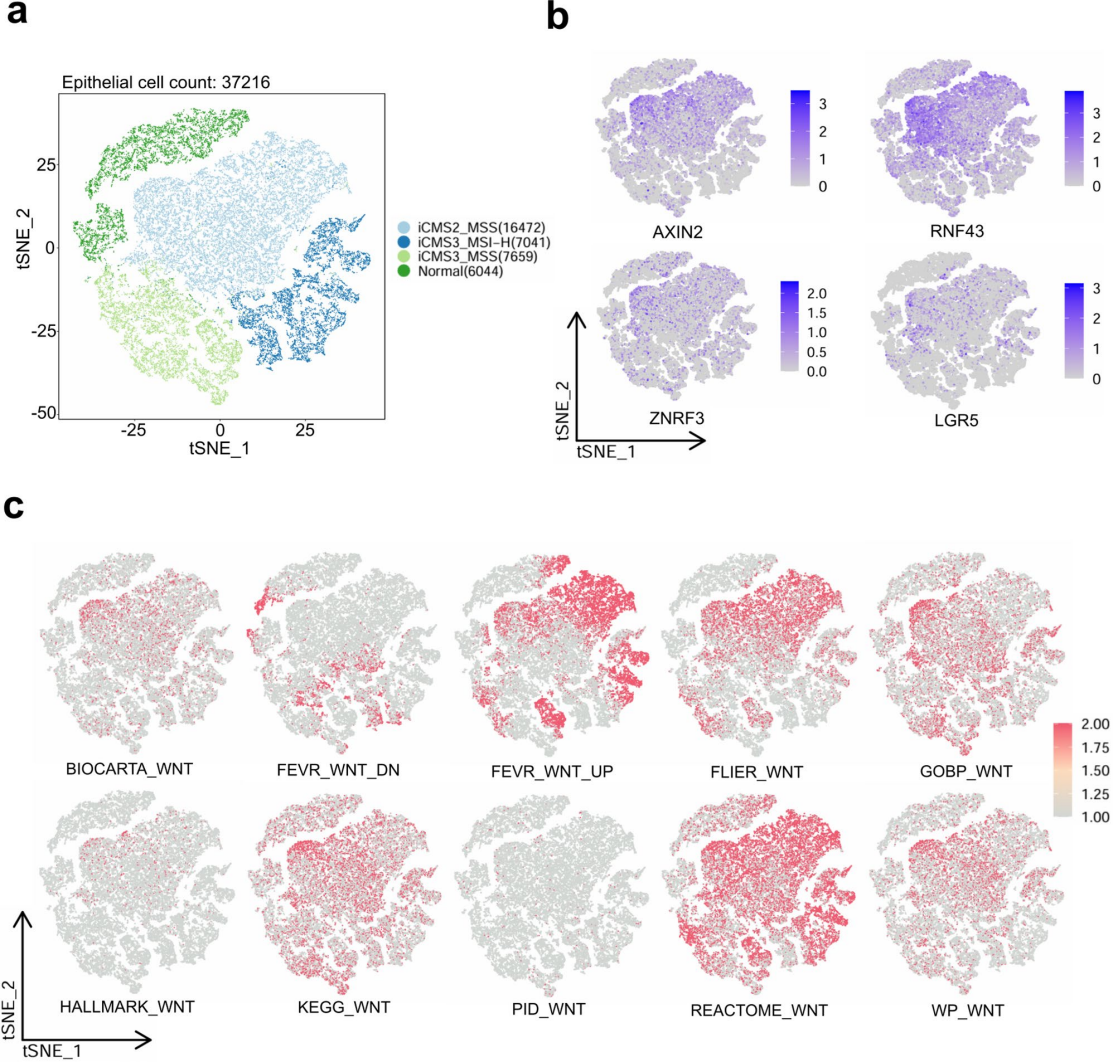
Analyses of single-cell data in CRC. (**a**) Two-dimensional t-SNE map of CRC single cell data. (**b**) Expression of marker genes of the Wnt/β-catenin signaling pathway. (**c**) AUCell enrichment scores for 10 candidate signatures

Single-cell RNA-Seq data, characterized by prominent dropout effects and technical noise, are well-suited for evaluating the robustness and validity of directional quantification. We collected a substantial single-cell RNA-seq dataset composed of 37, 216 CRC epithelial cells exhibited intratumoral heterogeneity [47], which could be classified with the iCMS single-cell subtyping system [46] into 16, 472 iCMS2 cells, 7, 041 microsatellite instability (MSI) iCMS3 cells, 7, 659 microsatellite stability (MSS) iCMS3 cells, as well as 6, 044 normal cells (Fig. 4a). As before, iCMS2 cells exhibited hyperactive Wnt signaling, and Wnt pathway markers such as AXIN2, RNF43, ZNRF3, and LGR5 were overexpressed in iCMS2 cells (Fig. 4b). By applying the AUCell algorithm, the ‘FEVR_WNT_DN’ signature was significantly reduced in iCMS2 cells and increased in iCMS3 or normal cells, revealing its suitability for characterizing Wnt pathway inhibition in single-cell data (Fig. 4c, Fig. S3a). In contrast, the ‘WP_WNT’, ‘HALLMARK_WNT’, and ‘FLIER_WNT’ signatures were significantly up-regulated in iCMS2 cells and down-regulated in iCMS3 or normal cells, effectively indicating Wnt pathway activation (Fig. 4c, Fig. S3a). Furthermore, density distribution analysis revealed that ‘FLIER_WNT’ was the only signature capable of correctly distinguishing Wnt activity within iCMS3 subtypes segregated by microsatellite instability (Fig. S3b-k). Overall, the performance of the these Wnt pathway signatures aligned with their ranking by MetaScreener in training datasets, especially the top positive signature ‘FLIER_WNT’ and the top negative signature ‘FEVR_WNT_DN’, confirming its capability of directionality establishment.

### Unsupervised directional analysis of established functional signatures in colorectal cancer

Beyond the supervised quantification of signature directionality, MetaScreener could also be applied in an unsupervised way without the training phase, which is useful for exploratory analysis given established gene signatures. Here, we sought to examine whether it could reveal biological insights by decoding the directional regulation of functional signatures in CRC. We systematically curated cancer hallmarks and CRC-related functions (Table S4) and analyzed their directional activities across 31 independent CRC datasets.

**Fig. 5:**
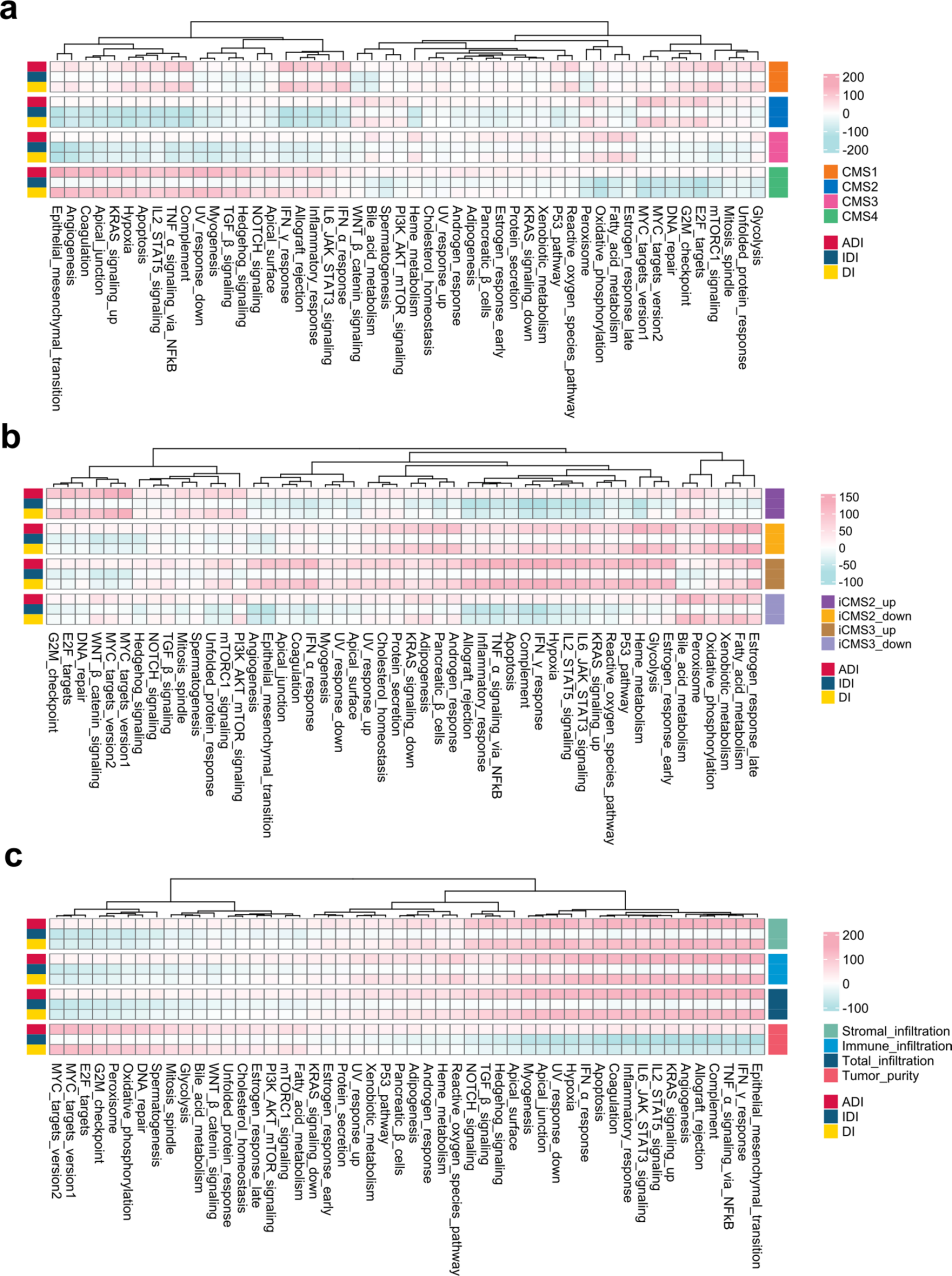
Directional scores for hallmark pathways in CRC subtypes. (**a**) Directionality indices of hallmark pathways in the CRC CMS subtyping system. (**b**) Directionality indices of hallmark pathways in the CRC iCMS subtyping system. (**c**) Directionality indices of hallmark pathways in relevance to the CRC tumor microenvironment using the ESTIMATE algorithm

CMS1, the immune activated subtype, demonstrated elevated ADI and DI scores for immune-related signatures, such as inflammatory response, IFN-α and IFN-γ responses, IL6-JAK-STAT3 signaling, and T/NK cell infiltration (Fig. 5a and Fig. S4a). CMS2, the hyperactive Wnt/MYC signaling subtype, showed high DI scores for canonical pathways such as Wnt, MYC, and E2F targets (Fig. 5a and Fig. S4a). CMS3, the metabolic dysregulation subtype, exhibited top ranked DI scores for fatty acid metabolism, oxidative phosphorylation, and relevant metabolites (Fig. 5a and Fig. S4a). CMS4, the mesenchymal subtype, harbored elevated DI scores for epithelial-mesenchymal transition (EMT), TGF-β signaling, and stromal infiltration (Fig. 5a and Fig. S4a).

Similar to CMS2, the iCMS2 subtype also exhibited hyperactive Wnt and MYC signatures (Fig. 5b and Fig. S4b). The iCMS3 subtype, on the other hand, showed activation of immune responses, inflammatory pathways, and EMT (Fig. 5b and Fig. S4b). These observations aligned with the study of Joanito et al. [46]. In addition, we also tested MetaScreener for delineating signatures in correlation to tumor infiltrations by stromal and/or immune fractions, which is inversely associated with tumor purity (Fig. 5c and Fig. S4c). Taken together, these clearly demonstrated the superior capability of MetaScreener in unsupervised directional analysis.

### Directional discovery of compounds targeting Wnt signaling and metastasis in colorectal cancer

**Fig. 6:**
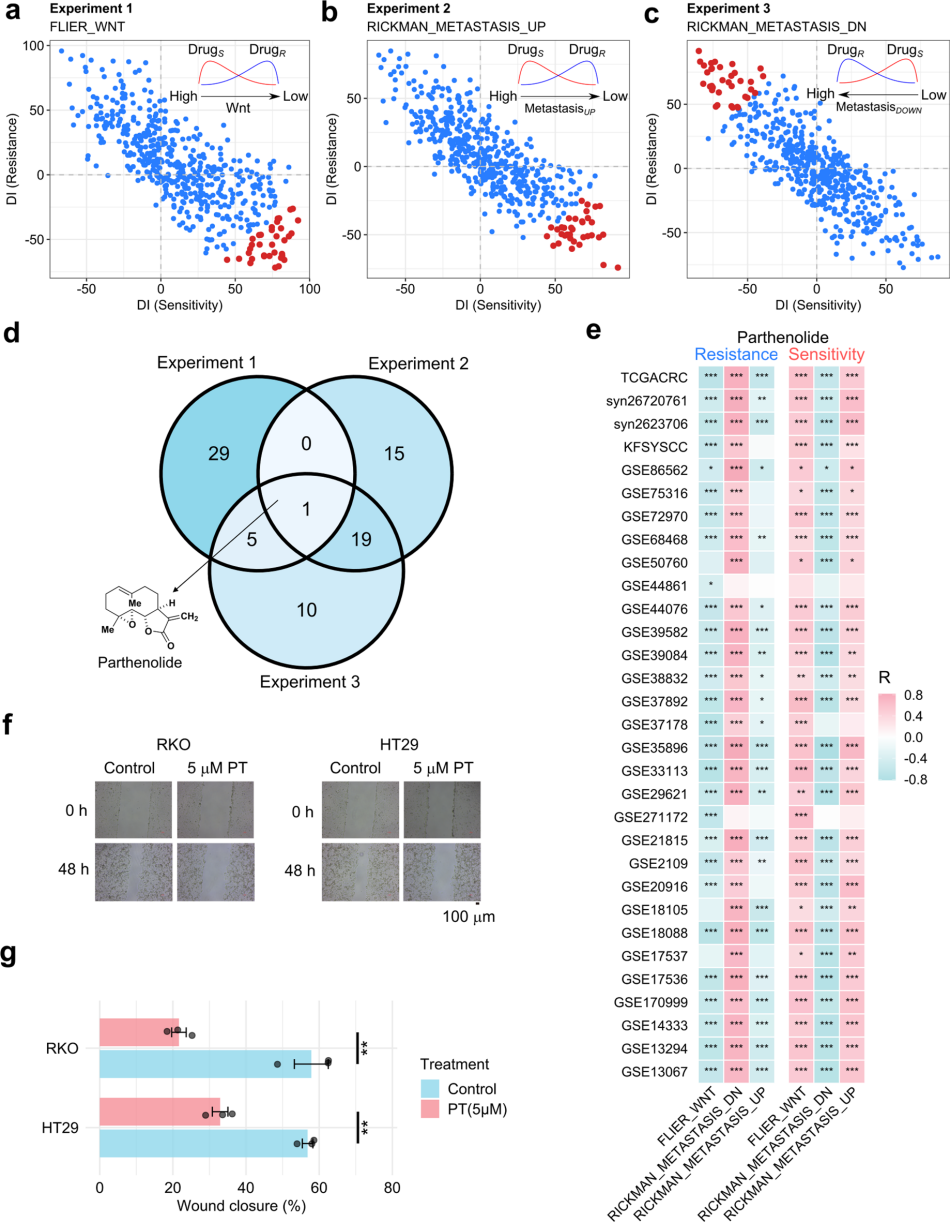
Signature-guided directional screening for compounds targeting Wnt pathway and metastasis in CRC. (**a**) Experiment 1: directional screening for drugs with a sensitivity signature Drug_*S*_ in positive correlation with the ‘FLIER_WNT’ signature, and vice versa for the resistance signature Drug_*R*_. That is, drugs killing Wnt hyperactive cells will be prioritized (red, top 35). Arrow direction: expected efficacy by drug (Wnt signaling from high to low). (**b**) Experiment 2: directional screening for drugs with Drug_*S*_ in positive correlation with the ‘RICKMAN_METASTASIS_UP’ signature, and vice versa for Drug_*R*_. That is, drugs killing migrative cells will be prioritized (red, top 35). Arrow direction: expected efficacy by drug (METASTASIS_UP from high to low). (**c**) Experiment 3: directional screening for drugs with Drug_*S*_ in negative correlation with the ‘RICKMAN_METASTASIS_DN’ signature, and vice versa for Drug_*R*_. That is, drugs not killing non-migrative cells will be prioritized (red, top 35). Arrow direction: expected efficacy by drug (METASTASIS_DN from low to high). (**d**) Venn diagrams summarizing the three drug screening experiments. The natural product parthenolide was the only consensus compound. (**e**) Correlation analysis of parthenolide resistance/sensitivity signatures with ‘FLIER_WNT’, ‘RICKMAN_METASTASIS_UP’, and ‘RICKMAN_METASTASIS_DN’ signatures in CRC datasets. * *p*-value < 0.05, ** *p*-value < 0.01, *** *p*-value < 0.001. (**f**) Wound healing assay of RKO and HT29 cells treated with or without 5 μM parthenolide (PT) for 48 hours. Microscopic images were taken at 0 and 48 hours post-scratching. (**g**) Wound closure of RKO and HT29 cells treated with or without 5 μM parthenolide (PT) for 48 hours. ** *p*-value < 0.01 (Student’s *t* test)

The directional characteristic of MetaScreener empowers drug signature screening to identify compounds with specific therapeutic functions. Given Wnt signaling as a well-known driver pathway in CRC, we sought to identify compounds that potentially target it. Meanwhile, we also require the compounds targeting cancer metastasis as well, so as to further narrow down drug candidates. We first compiled a compendium of 519 drugs from the CTRP project, each with CRC-specific sensitive (Drug_*S*_) and resistant (Drug_*R*_) signatures (Table S5). Subsequently, we designed three virtual directional CorMetaScreener screening experiments on the aforementioned 31 CRC datasets, with ssGSEA profiles of the ‘FLIER_WNT’ (Fig. 6a), ‘RICKMAN_METASTASIS_UP’ [48] (Fig. 6b), and ‘RICKMAN_METASTASIS_DN’ (Fig. 6c) signatures as continuous phenotypic states. The screening direction was for drugs killing Wnt hyperactive and metastatic cancer cells, while tolerating Wnt-negative or non-metastatic cells. Interestingly, parthenolide was the only candidate consistently ranking top in all the three experiments (Fig. 6d, Fig. S5, Table S6). Across the 31 CRC datasets, strong directional correlation patterns also supported the prioritization of parthenolide (Fig. 6e). Abundant reports further confirmed parthenolide as a Wnt signaling inhibitor [49, 50]. Therefore, we only experimentally tested its efficacy on metastasis inhibition in two CRC cell lines, RKO and HT29. Low concentrations of parthenolide significantly decreased the metastatic potential of both RKO and HT29 cells, an effect primarily attributed to the inhibition of cell migration capability rather than non-specific cytotoxicity (Fig. 6f–g, Fig. S6). Together with a relevant study in breast cancer [51], these indicate parthenolide as a potent inhibitor of metastasis. This case study further highlights the value of directional prioritization by MetaScreener. Additionally, we have also utilized the TCGA-CRC dataset to assess the computational speed of each individual method, thereby providing a basis for computationally efficient drug screening (Table S7).

## Discussion

We provide a comprehensive dual-mode framework for the prioritization of directional signatures given two types of training datasets (either DiffMetaScreener or CorMetaScreener mode). It can also work in an unsupervised manner for integrative scoring of pre-qualified signatures. Based on systematic assessments and case studies for CRC, we verify its noise-insensitivity, robustness and reliability, as well as a wide applicability in mechanistic studies and actionable reprogramming of cancer. Specifically, we discover parthenolide as a candidate drug that suppresses Wnt signaling and metastasis in aggressive CRC cells.

Our study reveals a serious lacking of directionality in commonly used pathways. The well-known Wnt signaling pathways, as defined in KEGG, GO, and REACTOME databases, turn out to be non-discriminative even in high-quality training datasets. One possibility is that they do not account for the directional activity of Wnt signaling, and merely contain a mixture of various functionally related genes, some of which might even be non-indicative of the pathway activity [52, 53]. According to our experience, such situation is not limited to the Wnt signaling pathway. Rather, it is highly prevalent in quite many functional ontologies and gene signatures. This status certainly fails to meet the prerequisites of precision medicine and actionable drug discovery via signature screening, as well as biomedical studies that heavily rely on gene signatures [54–56]. Moreover, it also threatens recent large-language model applications focusing on functional gene sets [57–59]. Given the huge number of gene sets (at the scale of 10, 000s) cataloged in functional databases, it is highly recommended to perform a pre-filtering with MetaScreener on some ‘minimalist’ training dataset(s), no matter bulk or single-cell. Even when no labels of definite activation/inhibition are available, the CorMetaScreener mode is still applicable as long as any correlated signatures are available for scoring and used as a reference (guilt-by-association). Hence, our framework is highly generalized and flexible for application to various types of data beyond bulk and single-cell transcriptomics, such as spatial transcriptomics [60], spatial proteomics [61], and metabolomics [62].

A critical challenge of oncology is to block cancer metastasis [63–65]. Wnt signaling, a canonical driver of CRC, also contributes to invasive metastasis through epithelial-to-mesenchymal transition (EMT) and microenvironment remodeling [66], although metastasis is a rather complex multistep process involving enormous factors. In addition, Wnt signaling regulates self-renewal and maintenance of cancer stem cells. Given the close connection and partial independence between Wnt signaling and metastasis, it would be interesting to evaluate simultaneous targeting of both functional aspects. As a proof of concept, we successfully discovered parthenolide as a potent dual inhibitor on both Wnt signaling and cancer cell migration, complementing structure-based multi-targeting approaches [67]. Based on our results and other reports [49, 50], future studies on parthenolide for Wnt hyperactive CRC therapeutics are highly warranted.

Traditional single-target inhibitors often fail due to compensatory mechanisms or tumor heterogeneity [68]. Addressing this challenge requires computational frameworks that predict patient responses to multi-targeted therapies, ultimately guiding more effective personalized treatment strategies [69]. MetaScreener, not only supports traditional single-target signature screening, but also demonstrates its capability for multiple-target signature screening. We designed three directional experiments to uncover parthenolide, rationalizing ‘killing two birds with one stone’ [70]. This application can be easily extended to more virtual experiments for large-scale combined prioritization of multi-targeting drugs. Such drug development strategies, not only coincide better with the heterogeneity and plasticity of cancer, but also increase drug response rates and delay acquired resistance. Although MetaScreener demonstrated robustness to Gaussian noise, biological variation in sequencing data may follow other distributions, such as the negative binomial. Therefore, validation under various noise models would be beneficial.

Beyond Wnt signaling, the challenge of directionality consistency extends to numerous well-established oncogenic pathways. For instance, pathways such as receptor-tyrosine kinase (RTK)/RAS/MAP-Kinase (MAPK) signaling, PI-3-Kinase (PI3K) signaling, p53 signaling, and cell cycle are frequently altered across diverse cancer types, and their transcriptional outputs often exhibit context-dependent variability [71]. Consequently, they often have various definitions across databases or literature. Together, these types of heterogeneity underscore the necessity of our DiffMetaScreener and CorMetaScreener frameworks, which are specifically designed to systematically evaluate and reconcile directional inconsistencies across multiple contexts, thereby enabling robust and reproducible signature-based discoveries in complex oncogenic landscapes.

## Materials and methods

### Collection of datasets and signatures

The CRC training datasets were retrieved through Gene Expression Omnibus (GEO, https://www.ncbi.nlm.nih.gov/geo/), Synapse (https://www.synapse.org/), and ‘TCGAbiolinks’ package [72], with the following accession numbers: GSE33113, GSE39582, GSE35896, GSE13067, GSE13294, GSE14333, GSE20916, TCGA-CRC (combined from TCGA-COAD and TCGA-READ), and KFSYSCC (syn4974668). The independent CRC validation datasets were similarly retrieved with the following accession numbers: GSE44076, GSE44861, GSE68468, GSE37178, GSE18105, GSE21815, GSE17537, GSE29621, GSE38832, GSE72970, GSE2109, GSE17536, GSE37892, syn26720761, syn2623706 (multiple datasets de-batched and integrated, see reference study [45] for details.), GSE39084, GSE170999, GSE18088, GSE75316, GSE271172, GSE50760, and GSE86562. The Wnt pathway signatures used for screening were obtained from MSigDB [15] (https://www.gsea-msigdb.org/) and publications [45]. As some signatures did not consistently use current official gene symbols, we performed manual updates using GeneCards (https://www.genecards.org/). See Table S1-2 for a compendium of all dataset used and Table S3 for all the gene signatures used.

### Construction of a dual-mode screening framework

The screening framework was comprised of two distinct running modes: DiffMetaScreener for differential enrichment analysis and CorMetaScreener for correlative enrichment analysis.

DiffMetaScreener integrated 23 signature enrichment approaches: GSVA, ssGSEA, Z-score, and PLAGE using the ‘GSVA’ package [20], PCA-based enrichment (‘IOBR’ package) [73], AUCell and UCell analyses [24, 25], Singscore analysis (‘singscore’ package) [26], Median enrichment calculated as the median expression value of genes within each signature, as well as eleven methods included in the ‘decoupleR’ package [27] multivariate decision trees (MDT), multivariate linear model (MLM), univariate decision tree (UDT), univariate linear model (ULM), virtual inference of protein-activity by enriched regulon (VIPER), weighted mean (WMEAN), norm_WMEAN (permutation-based normalized enrichment scores), corr_WMEAN (permutation-based corrected enrichment scores), weighted sum (WSUM), norm_WSUM (permutation-based normalized enrichment scores), and corr_WSUM (permutation-based corrected enrichment scores), and two group-level enrichment analysis methods based on the gene-level differential expression results, with -log_10_(*p*-value) × log_2_FC used as the ranking statistic for FGSEA (‘fgsea’ package), and top 1000 up- and down-regulated genes (ranking by *p*-values) used for ORA (‘clusterProfiler’ package) [74]. Single-sample signature scores were tested for differential activities in respect to the ‘activation/inhibition’ labels using six approaches (limma [75], Student’s t-test, ANOVA, Wilcoxon rank-sum test, Kruskal-Wallis rank sum test, and permutation test [76]) to derive two-tailed *p*-values and directional statistics. Next, all two-tailed *p*-values were approximately converted to directional one-sided *p*-values with the ‘two2one’ function in the ‘metap’ package, and subsequently combined using 10 methods from the ‘metap’ package (‘invt’, ‘invchisq’, ‘logitp’, ‘meanp’, ‘meanz’, ‘sumlog’, ‘sumz’, ‘sump’, ‘votep’, and ‘wilkinsonp’), plus another two methods, the Cauchy combination test (CCT) [77] and the geometric mean. Notice that these methods were for combining within-dataset comparisons, and for cross-dataset comparisons only geometric mean was used. The combined positive and negative directional *p*-values were transformed through -log_10_ and log_10_ transformations respectively, yielding activation directionality indices (ADI, all were positive) and inhibition directionality indices (IDI, all were negative) for each of the twelve *p*-value combination methods. Subsequent averaging produced the final ADI and IDI, as well as the final directionality indices (DI) as the net sum of ADI and IDI.

CorMetaScreener, although philosophically similar to DiffMetaScreener, occupied several unique features: (1) continuous phenotypic state variables instead of discrete ‘activation/inhibition’ group labels, and (2) phenotypic relevance quantified with correlation analysis methods rather than differential analysis methods. Among the correlation methods, Pearson, Kendall, Spearman, and linear models (LM) were implemented in the ‘stats’ package, while Biweight, Distance, Percentage, Blomqvist, Hoeffding, and Gamma were from the ‘correlation’ package. For FGSEA, -log_10_(*p*-value) × *r* (where *r* represents the correlation coefficient, or the slope *β* in LM) was taken as the ranking statistic. For ORA, top up- and down-regulated genes were ranked by the magnitude of *r* (or *β* in LM) on each direction, respectively.

### Evaluation of reliability

For reliability evaluation, we took the heterogeneity of the Wnt/β-catenin pathway in CRC as a case study. CRC has 2 dominant molecular subtyping systems: the CMS (consensus molecular subtypes) system (CMS1, CMS2, CMS3, and CMS4) [45] and the iCMS (intrinsic consensus molecular subtypes) system (iCMS2 and iCMS3) [46], with CMS2 and iCMS2 representing the Wnt hyperactive subtypes in their respective systems. Thus, we designed 2 experimental approaches: (1) Discrete CMS subtype labels were determined by the CMS classification as implemented in the ‘CMSclassifier’ package [45] for nine CRC datasets (Table S2), and after discarding samples labeled as ‘NOLBL (unclassified samples)’, the CMS2 samples were denoted as the Wnt ‘activation’ group, and the CMS1/3/4 samples the ‘inhibition’ group. DiffMetaScreener mode analysis was performed on 10 candidate Wnt pathway signatures. Independent validation was evaluated with ssGSEA and Wilcoxon rank-sum test in 22 testing datasets. (2) Continuous phenotypic scores of the same training samples in nine datasets were obtained by ssGSEA quantification on an iCMS2-upregulated signature (‘iCMS2_up’) [46] (Table S3). CorMetaScreener mode analysis was performed on the same 10 candidate Wnt pathway signatures. Independent validation was evaluated with Pearson correlation coefficients between the ssGSEA activity profiles of these 10 signatures and the ‘iCMS2_up’ signature scores.

### Evaluation of robustness

For robustness evaluation, we designed 7 experimental scenarios to comprehensively assess DiffMetaScreener and CorMetaScreener: (1) Random exclusion of three training datasets in nine (30 repetitions); (2) Random removal of 30% genes from training datasets (30 repetitions); (3) Introduction of random Gaussian noise matrices (30 repetitions, zero-mean and randomized standard deviation in [0.05, 0.2]), with varying noise proportions (0% to 100%) and types (15 additive and 15 multiplicative) and non-negativity enforced (zero-thresholding); (4) Random elimination of 30% of enrichment analysis methods (30 repetitions); (5) Random removal of four in the twelve *p*-value combination methods (30 repetitions); (6) Executing per-method analyses for all enrichment methods (138 cycles for DiffMetaScreener and 230 cycles for CorMetaScreener) while recording the runtime of each method specifically on the TCGA-CRC dataset (Table S7); (7) Performing per-method evaluations for all *p*-value combination methods (12 cycles each). In each of these scenarios, Pearson’s correlation coefficients were used to determine the consistency of the perturbated DI and the original DI.

For extreme scenarios of noise and information loss, the perturbations were designed as follows: 70% of the genes in the input dataset were randomly removed, followed by the introduction of strong Gaussian noise (mean = 1, random standard deviation ranging from 1 to 2) at a high noise ratio (50% − 100%), again including 15 types of additive and multiplicative noises respectively plus zero-thresholding to ensure non-negativity. In contrast, under traditional single-dataset and single-enrichment applications, a single dataset was randomly selected from the training data, and each enrichment method was evaluated sequentially, with 30 repetitions per method. Pearson’s correlation coefficients were again used for robustness comparison.

### Single-cell RNA-seq data analyses

The single-cell RNA sequencing data of CRC epithelial cells were obtained from Synapse (syn26844071) [46], comprising five CRC batches (CRC16, JSC, KUL3, KUL5, SMC). Doublets were detected and removed using the ‘scDblFinder’ package [78], followed by quality control with the ‘Seurat’ package [79], retaining cells with 200 to 7500 unique genes and excluding low-quality cells with mitochondrial gene content over 10%. Highly variable genes (HVGs) were selected using the ‘FindVariableGenes’ function in ‘Seurat’, and data normalization was performed via the ‘ScaleData’ function. PCA analysis was conducted based on these HVGs, and batch effects were corrected using the ‘harmony’ package. A five-dimensional group-aware embedding was generated using the PLS-DA algorithm (‘mixOmics’ package) [80], which was subjected to t-SNE dimensionality reduction and visualization (‘SCP’ package). AUCell activity scoring and visualization for gene signatures were performed using the ‘irGSEA’ package [81], with the sparsity threshold parameter ‘aucell.MaxRank’ set to the default of the top 5% of genes.

### Unsupervised-screening-based empirical assessment using biological signatures

A compendium of 50 cancer hallmarks [17] from MSigDB [15] were broadly categorized. Meanwhile, we curated another 50 CRC-related signatures from publication [45]. A full list of these signatures was shown in Table S4.

These signatures were analyzed with DiffMetaScreener in a total of 31 CRC datasets (training and independent validation datasets, Tables S1-2) using four comparisons by labeling ‘activation/inhibition’ to the following groups: CMS1 versus Others, CMS2 versus Others, CMS3 versus Others, and CMS4 versus Others. Similarly, they were also analyzed with CorMetaScreener by correlating to the iCMS2 up/downregulated (‘iCMS2_up’ and ‘iCMS2_down’) and iCMS3 up/downregulated (‘iCMS3_up’ and ‘iCMS2_down’) [46] signature scores. Furthermore, we employed the ESTIMATE algorithm [82] to calculate stromal scores, immune scores, ESTIMATE scores, and tumor purity for each of the 31 CRC datasets individually.

### Signature-guided directional drug discovery

MetaScreener can be applied in screening drugs for specific therapeutic effects. Drug sensitivity/resistance data were downloaded as cell line gene expression matrices and drug sensitivity profiles from the CTRP project via OSF (https://osf.io/c6tfx/files/osfstorage) [83]. To ensure context specificity and accuracy, non-CRC cell lines were excluded, and only drugs with valid sensitivity measurements in ≥ 12 CRC cell lines were retained, yielding 519 compounds. For each drug, cell lines were ranked by descending IC50 values and stratified into three groups as follows: top 25% as highly resistant, bottom 25% as highly sensitive, and the remaining 50% as intermediate. Notice this is not classified by thresholds but by proportions in the cell line ranking. Differential expression analysis (‘limma’ package [75]) of high-resistance versus high-sensitivity groups resulted in top 100 up- and down-regulated genes (ranked by ascending *p*-values) respectively, which were extracted as drug-specific sensitivity/resistance gene signatures, designated as Drug_*S*_ and Drug_*R*_, respectively (519 signature pairs per drug). See Table S5 for a list of these drug gene signature pairs.

In this case study, MetaScreener was utilized to prioritize drugs that simultaneously inhibit Wnt pathway activity and suppress CRC metastasis. We designed three screening experiments with CorMetaScreener to rank drugs by quantifying their Drug_*S*_ and Drug_*R*_ signature pairs with respect to: (1) ranking by correlation to the ‘FLIER_WNT’ ssGSEA signature scores, (2) ranking by correlation to the ‘RICKMAN_METASTASIS_UP’ [48] ssGSEA signature scores, and (3) ranking by correlation to ‘RICKMAN_METASTASIS_DN’ ssGSEA signature scores. Since the purpose was to discover drugs inhibiting Wnt pathway and CRC migration, the Drug_*S*_ DI scores should be positively correlated with ‘FLIER_WNT’ and ‘RICKMAN_METASTASIS_UP’, and negatively correlated with ‘RICKMAN_METASTASIS_DN’, and vice versa for Drug_*R*_. See also Figure 6 (a-c) for a visual demonstration. The configuration of CorMetaScreener running included 3 unsupervised enrichment methods (GSVA, ssGSEA, and Z-score) and 3 correlation methods (Pearson, Spearman, and Kendall). Venn diagrams were created with the ‘ggVennDiagram’ package. See Table S6 for all screening results.

### Cell scratch assay

To perform the wound healing assay, RKO and HT29 cells were grown-up to ~80% confluence in 6-well plates. A straight scratch was made slowly across the center of the well by a P200 pipette tip. Detached cells were washed away with phosphate buffered saline (PBS) and added serum-free medium with a low concentration of parthenolide (5μM). Cell migration to scratch was monitored at the indicated time periods in the same area. The capability of the cells to close the wounds, and cell motility, were evaluated by determining the healed area. The images were taken. The measured time points were 0 hours and 48 hours after treated by parthenolide. The experiments were repeated three times. Student’s *t* test was employed to evaluate the statistical significance of wound closure percentages.

### Cell viability assay

For cell viability assays, cells in log-phase were seeded in 96-well plates (800 cells/well) for 24 h and then treated with 5 μM parthenolide. After 48 h, 10 μl of CCK-8 reagent was added to each well and the plates were incubated for 1 h at 37 °C. The absorbance was measured in single-wavelength mode (450 nm). The experiments were repeated four times. Student’s *t* test was employed to evaluate the statistical significance of cell viability.

## Electronic supplementary material.

Below is the link to the electronic supplementary material.

Supplementary material 1

Supplementary material 2

Supplementary material 3

Supplementary material 4

Supplementary material 5

Supplementary material 6

Supplementary material 7

Supplementary material 8

## Data Availability

All dataset accessions, signatures, and data files are provided in the supplementary tables. All the codes are in the accompanied GitHub repository (https://github.com/FangZY-Lab/MetaScreener).
