# Supplementary material for: MetaScreener: a robust dual-mode framework for directional prioritization of actionable signatures through multi-dataset and multi-approach integration
Source: J Transl Med. 2026 Mar 17;24:583. doi: 10.1186/s12967-026-08019-y (PMC13107662; doi:10.1186/s12967-026-08019-y)
Supplement: Supplementary file 8 — Supplementary material 8 [file 12967_2026_8019_MOESM8_ESM.pdf]

Supplementary Data for "MetaScreener: a robust dual-mode framework for directional prioritization of actionable signatures through multi-dataset and multi-approach integration"

Dingkang Zhao [1, 2, #], Gaoxiang Zhao [3, #], Minghui Yao [1, 2], Jianxiong Wu [1, 2], Zhaoyuan Fang [1, 2, *]

[1] Department of Colorectal Surgery and Oncology, the Second Affiliated Hospital, and Center for Biomedical Systems and Informatics, Zhejiang University-University of Edinburgh Institute (ZJU-UoE Institute), Zhejiang University School of Medicine, Zhejiang University, Hangzhou 310000, Zhejiang, China.

[2] Edinburgh Medical School: Biomedical Sciences, College of Medicine and Veterinary Medicine, University of Edinburgh, Edinburgh EH8 9YL, Scotland, United Kingdom.

[3] Key Laboratory of Tropical Translational Medicine of Ministry of Education, School of Basic Medicine and Life Sciences, Hainan Medical University, Haikou 571199, Hainan, China.

[#] Equal contribution

[*]Corresponding author: Zhaoyuan Fang (fangzhaoyuan@sibs.ac.cn;zhaoyuanfang@intl.zju.edu.cn)

Supplementary Tables

Table S1. Colorectal cancer (CRC) training and independent validation datasets with references.

Table S2. Summary of CMS subtyping for CRC training and independent validation datasets.

Table S3. Candidate Wnt signaling signatures and iCMS up-/down-regulated signatures.

Table S4. Hallmark signatures and CRC-related signatures.

Table S5. Drug sensitivity/resistance signatures. Drug-resistance and -sensitive signatures are denoted by the suffix ‘UP’ and ‘DN’, respectively. Data source: CTRP.

Table S6. Summary of drug screening results. Metastasis signatures are also included here.

Table S7. Computational time for per-method analyses in the TCGA-CRC dataset.

Supplementary Figures and Legends

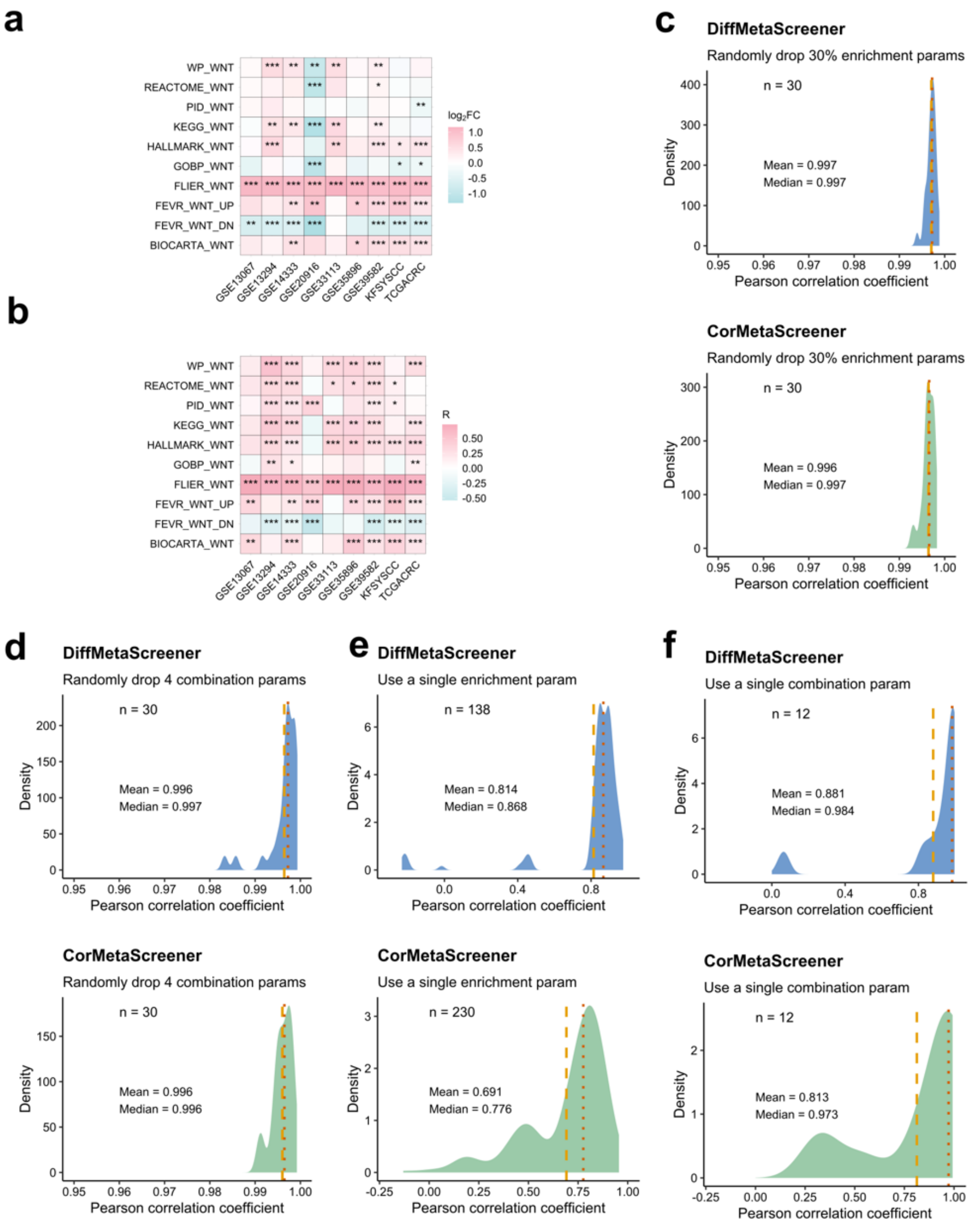

Figure S1. Additional assessment of MetaScreener on the 9 CRC training datasets.

(a) Qualitative visualization of signature directionality based on ssGSEA and differential enrichment analysis. * *p*-value < 0.05, ** *p*-value < 0.01, *** *p*-value < 0.001.

(b) Qualitative visualization of signature directionality based on ssGSEA and correlative enrichment analysis. * *p*-value < 0.05, ** *p*-value < 0.01, *** *p*-value < 0.001.

(c) Robustness testing for randomly deleting 30% of the enrichment methods. Repeat 30 times.

(d) Robustness testing for random deletion of 4 *p*-value combination methods. Repeat 30 times.

(e) Robustness testing performed on a per-method basis for enrichment methods.

(f) Robustness testing performed on a per-method basis for *p*-value combination methods.

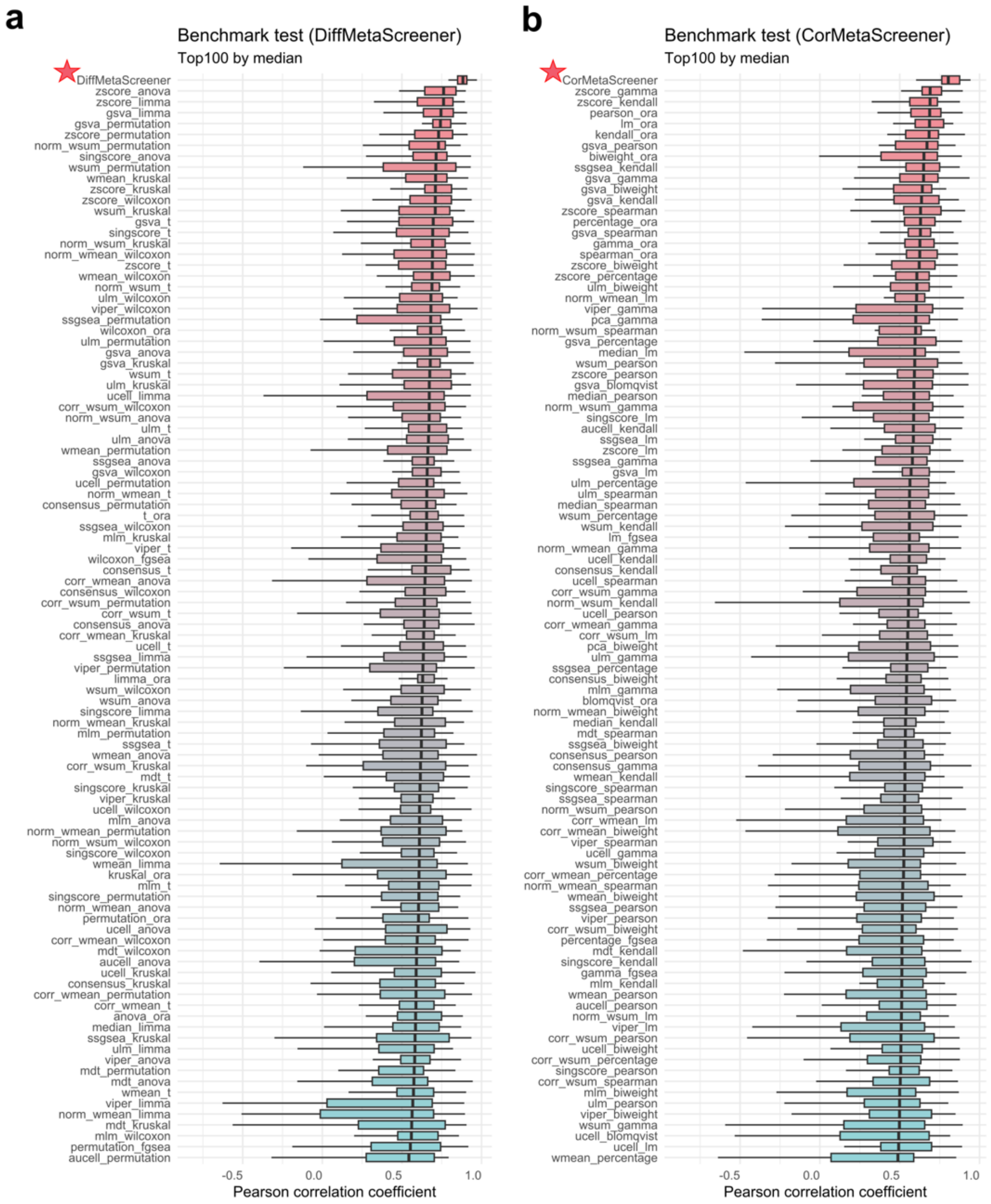

Figure S2. Benchmarking robustness of MetaScreener on 9 CRC training datasets.

(a) Robustness of DiffMetaScreener and conventional approaches under extreme noise. Top 100 approaches by median Pearson correlation were shown. Random deletion of 70% of the genes was accompanied by the introduction of strong noise (mean = 1, standard deviations ranging from 1 - 2), and the proportion of noise was 50% - 100%. Repeat 30 times (15 times each for additive and multiplicative noise).

(b) Robustness of CorMetaScreener and conventional approaches under extreme noise. Top 100 approaches by median Pearson correlation were shown. Random deletion of 70% of the genes was accompanied by the introduction of strong noise (mean = 1, standard deviations ranging from 1 - 2), and the proportion of noise was 50% - 100%. Repeat 30 times (15 times each for additive and multiplicative noise).

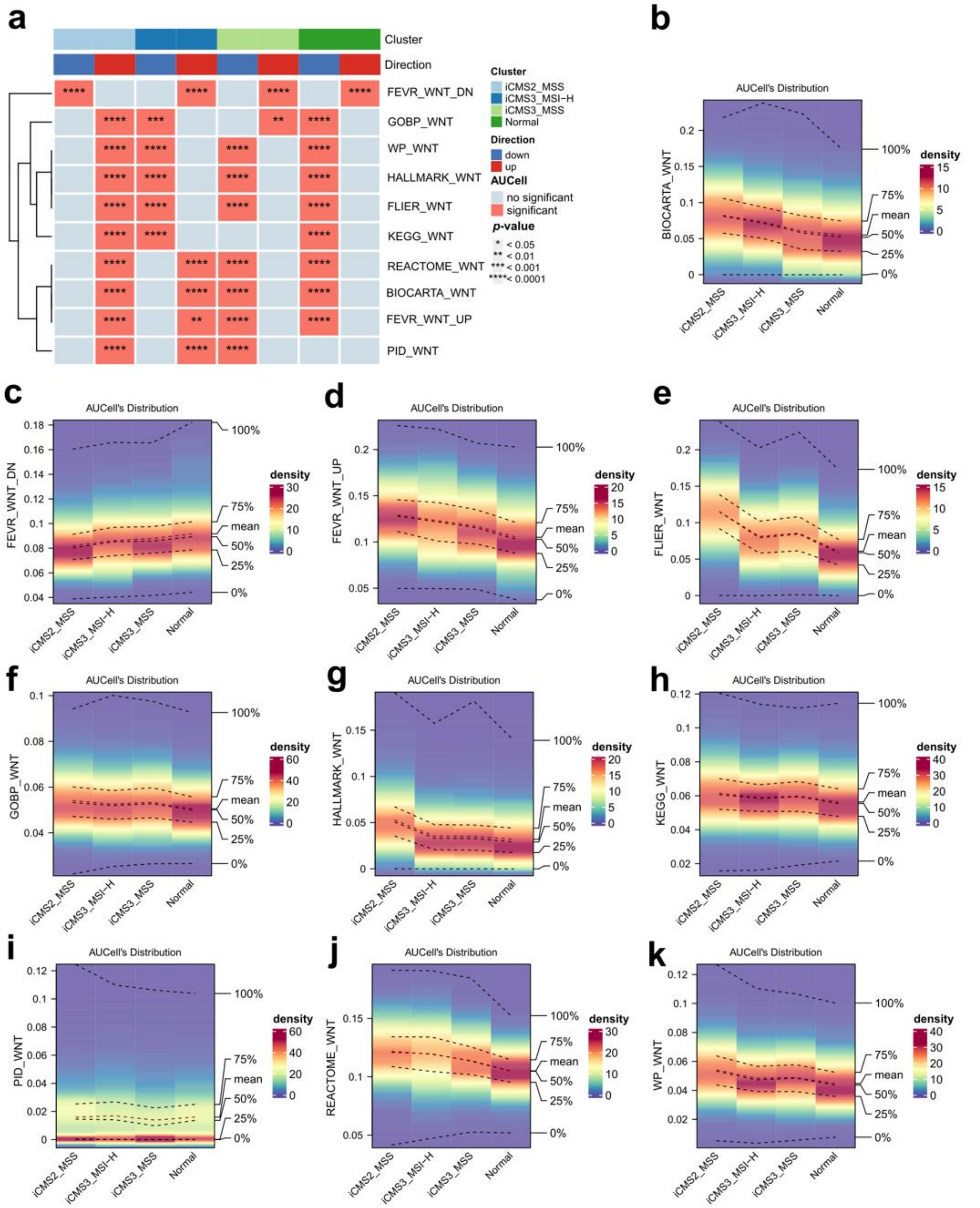

Figure S3. Additional AUCell analyses of the single-cell dataset in CRC.

(a) The differential (up/down) AUCell activities of candidate Wnt signatures across iCMS cell clusters. ** *p*-value < 0.01, **** *p*-value < 0.0001.

(b-k) Distribution of AUCell activities of candidate Wnt signatures across cell clusters. Candidate signatures include: ‘BIOCARTA_WNT’**(b)**, ‘FEVR_WNT_DN’**(c)**, ‘FEVR_WNT_UP’**(d)**, ‘FLIER_WNT’**(e)**, ‘GOBP_WNT’**(f)**, ‘HALLMARK_WNT’**(g)**, ‘KEGG_WNT’**(h)**, ‘PID_WNT’**(i)**, ‘REACTOME_WNT’**(j)**, and ‘WP_WNT’**(k)**.

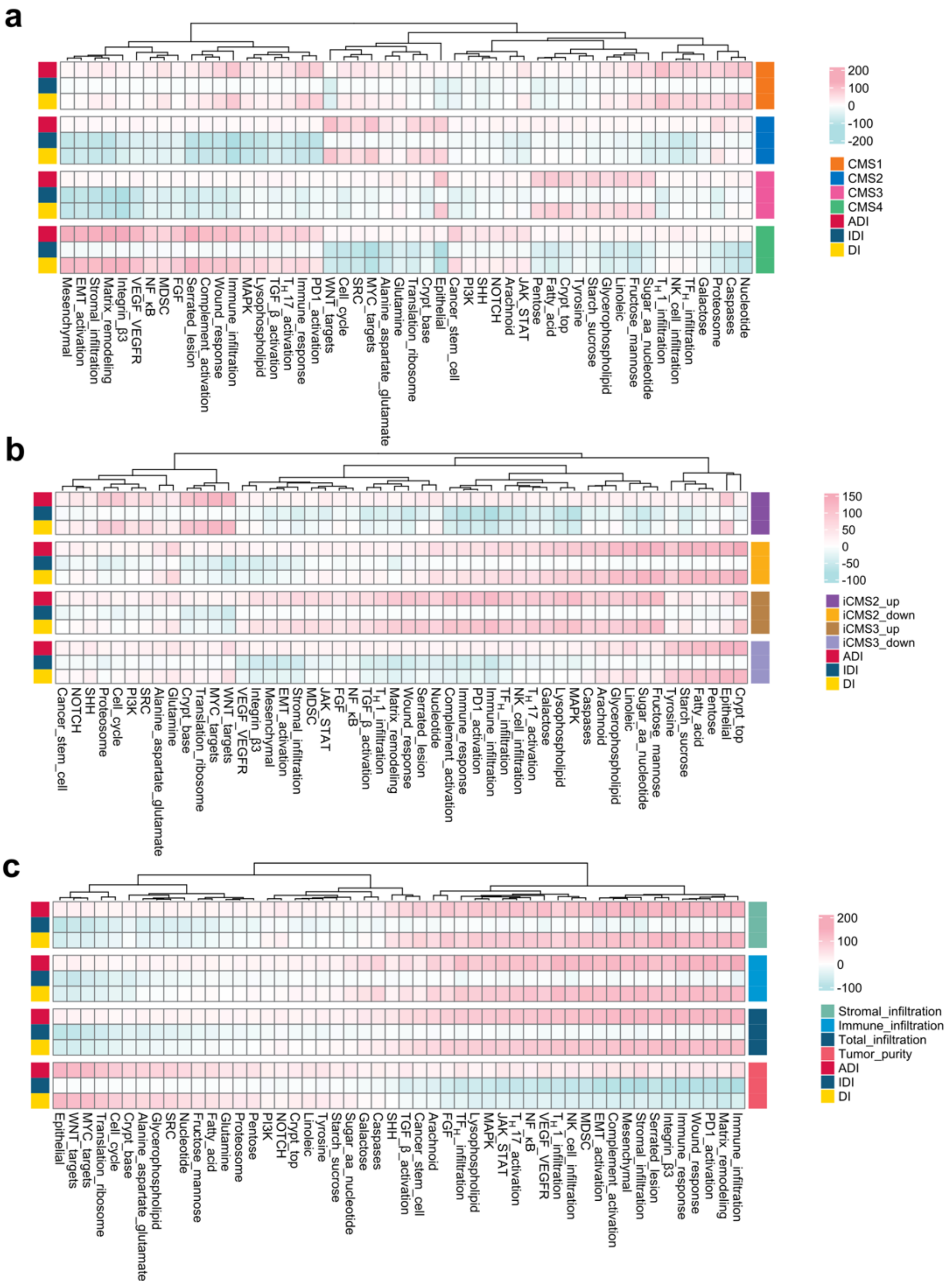

Figure S4. Directional scores for CRC-related pathways in CRC subtypes.

(a) Directionality indices of CRC-related signatures in the CMS subtyping system.

(b) Directionality indices of CRC-related signatures in the iCMS subtyping system.

(c) Directionality indices of CRC-related signatures in relevance to the CRC tumor microenvironment using the ESTIMATE algorithm.

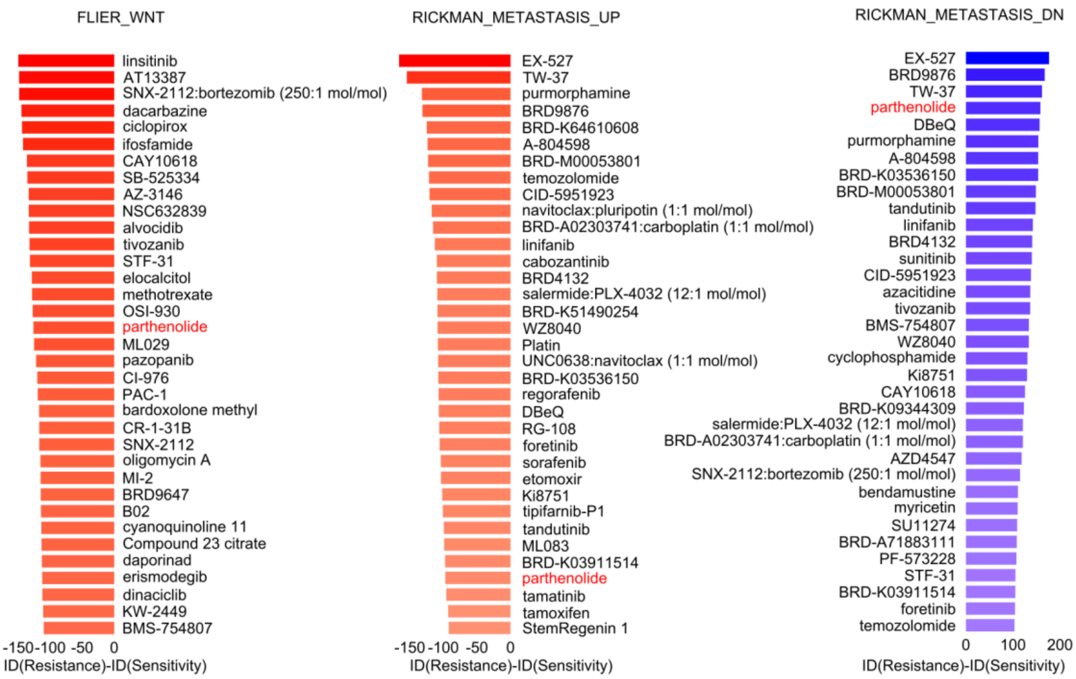

Figure S5. Identification of candidate drugs with dual inhibition on Wnt signaling and metastasis in CRC.

Top 35 drugs were shown for each experiment (‘FLIER_WNT’, ‘RICKMAN_METASTASIS_UP’, and ‘RICKMAN_METASTASIS_DN’). Parthenolide, a drug common to all three screening experiments, is shown in red.

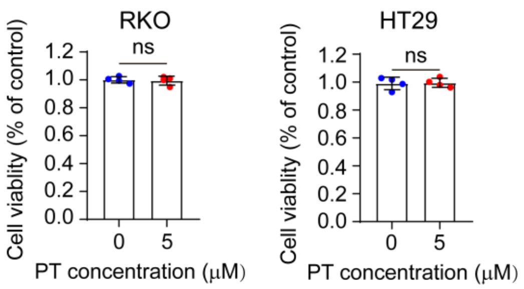

Figure S6. Cell viability assays in colorectal cancer cell lines.

RKO and HT29 cells treated with or without 5 μM parthenolide (PT) for 48 hours. ns: p-value > 0.05 (Student's t test).
